# HETEROGENEITY OF SENESCENT RIBOSOME COMPLEX AFFECTS THE TRANSLATIONAL EFFICIENCY OF SENESCENCE RELATED MRNAS

**DOI:** 10.1093/geroni/igz038.376

**Published:** 2019-11-08

**Authors:** Joon Kim, Hag Dong Kim, Hee Woong Yang

**Affiliations:** 1 Korea University, Seoul, Korea, Republic of; 2 HAEL Lab, Seoul, Korea, Republic of

## Abstract

The ribosome, a protein factory, has a lateral stalk known as the ribosomal P complex made up of rpLP0, rpLP1, and rpLP2. It plays an important role in translation by recruiting translational factors. One of these proteins, rpLP2, was decreased in translating ribosome when cellular senescence was induced. Additionally, Y-box binding protein-1 (YB-1), a multifunctional protein that regulates the transcription and translation, was also reduced in polysomal fraction of senescent cells. We have discovered that rpLP2 depletion in heterogeneous ribosome causes the detachment of YB-1 in polysomes and link to cellular senescence. Here, we also have found that a decrement of CK2α or GRK2 on senescent cells induced an increment of unphosphorylated rpLP2, resulting in the release of YB-1 from a ribosome complex. The heterogeneous senescent ribosome has different translational efficiency for some senescence related genes such as AHR, RAB27B, FEZ1, and DDIT4. Our results revealed that the decrease of rpLP1/rpLP2 and YB-1 in translating senescent ribosomes is not specific to cell type or stress type. Furthermore, the same phenomenon was observed in aged mouse liver. Taken together, our results suggest that the senescent ribosome complex appears to have low levels of rpLP1/ rpLP2 and YB-1, resulting in the alteration of translational efficiency for senescence related genes. (Journals of Gerontology: Biological Sciences, 2019 in press)

